# The Receptor-Like Kinase *ERECTA* Confers Improved Water Use Efficiency and Drought Tolerance to Poplar via Modulating Stomatal Density

**DOI:** 10.3390/ijms22147245

**Published:** 2021-07-06

**Authors:** Huiguang Li, Yanli Yang, Houling Wang, Sha Liu, Fuli Jia, Yanyan Su, Shuang Li, Fang He, Conghua Feng, Mengxue Niu, Jie Wang, Chao Liu, Weilun Yin, Xinli Xia

**Affiliations:** Beijing Advanced Innovation Center for Tree Breeding by Molecular Design, College of Biological Sciences and Technology, National Engineering Laboratory of Tree Breeding, Beijing Forestry University, Beijing 100083, China; hg_li@bjfu.edu.cn (H.L.); yangyl@bjfu.edu.cn (Y.Y.); whling@bjfu.edu.cn (H.W.); liushassl@163.com (S.L.); fulijia1219@163.com (F.J.); suyanyan5566@163.com (Y.S.); v_lishuang1@163.com (S.L.); hefang14686@sicau.edu.cn (F.H.); fengconghua2020@163.com (C.F.); niumengxue@bjfu.edu.cn (M.N.); wangjie@bjfu.edu.cn (J.W.); liuchao1306@bjfu.edu.cn (C.L.); yinwl@bjfu.edu.cn (W.Y.)

**Keywords:** *Populus*, *ERECTA*, stomatal density, water use efficiency, drought stress tolerance

## Abstract

Poplar is one of the most important tree species in the north temperate zone, but poplar plantations are quite water intensive. We report here that CaMV 35S promoter-driven overexpression of the *PdERECTA* gene, which is a member of the LRR-RLKs family from *Populus nigra* × (*Populus deltoides* × *Populus nigra*), improves water use efficiency and enhances drought tolerance in triploid white poplar. *PdERECTA* localizes to the plasma membrane. Overexpression plants showed lower stomatal density and larger stomatal size. The abaxial stomatal density was 24–34% lower and the stomatal size was 12–14% larger in overexpression lines. Reduced stomatal density led to a sharp restriction of transpiration, which was about 18–35% lower than the control line, and instantaneous water use efficiency was around 14–63% higher in overexpression lines under different conditions. These phenotypic changes led to increased drought tolerance. *PdERECTA* overexpression plants not only survived longer after stopping watering but also performed better when supplied with limited water, as they had better physical and photosynthesis conditions, faster growth rate, and higher biomass accumulation. Taken together, our data suggest that *PdERECTA* can alter the development pattern of stomata to reduce stomatal density, which then restricts water consumption, conferring enhanced drought tolerance to poplar. This makes *PdERECTA* trees promising candidates for establishing more water use efficient plantations.

## 1. Introduction

Drought stress is one of the most destructive agriculture calamities, as it penalizes the growth and distribution of plants, leading to economic loss and ecological damage [1,2]. One of the effective strategies to enhance drought tolerance is limiting water loss through transpiration [3,4]. Stomatal density, hormones, nutrition, blue light, humidity, or CO_2_ concentrations can affect plant water consumption, mainly by directly impacting stomatal conductance and transpiration rate [5,6,7,8,9,10,11,12,13], or mitigating the impairment of the plant cells brought by water depletion [14]. In recent decades, many reports demonstrated that manipulating critical genes can be a practical way to regulate water consumption [15,16,17,18,19].

Stomata are microscopic pores encompassed by a pair of guard cells, through which most terrestrial plants uptake CO_2_ for photosynthesis and discharge water vapor and O_2_ [20]. Under short-term drought stress, plants can restrict water loss via simply regulating stomatal apertures temporarily by hormones, such as abscisic acid (ABA) and brassinosteroids [21,22,23], while when exposed to long-term drought stress, plants reduce the stomatal density or shrink the leaf area permanently to restrict water consumption [3,17,24,25]. Mature leaves are sensors of the changing conditions of the environment, as they can generate signals and transport to developing leaves to regulate the formation of stomata in young leaves [26,27]. The formation of mature stomata in *Arabidopsis* undergoes several intermediary steps, which is known as the stomatal lineage, from the meristemoid cell (MMC) to the stomatal lineage ground cell (SLGC), the round guard mother cell (GMC), and at last the guard cells (GCs) [20,28]. Three bHLH-type transcription factors, *SPEECHLESS* (*SPCH*), *MUTE*, and *FAMA*, are fateful for these transformations [28,29,30].

Receptor-like kinases (RLKs) are one of the largest families containing versatile N-terminal extracellular domains and C-terminal intracellular kinases, which participate in receiving and conducting a wide range of signals or stimuli [31]. LRR-RLKs (leucine-rich repeats receptor-like kinases) are the largest group of RLKs composed of three distinct domains: a leucine-rich repeat (LRR) extracellular domain to perceive signals, a transmembrane region to anchor the protein within the membrane, and a cytoplasmic serine/threonine (Ser/Thr) protein kinase domain to transduce the signal downstream [32,33]. LRR-RLKs control a wide range of physiological responses in plants, not only in growth and development but also in responding to abiotic and biotic stresses [32,34,35]. As part of the LRR-RLKs family, *ERECTA* encodes a leucine-rich repeat receptor kinase [36]. Together with its two homologous genes, *ER-like 1* (*ERL1*) and *ERL2*, *ERECTA* plays a crucial role in the process of the stomata development signaling pathway. *ERECTA* is predominantly expressed in the shoot apical and organ primordia [37,38], and has a great impact on plant morphogenesis [39,40,41,42] by regulating phytohormones [38,43,44,45,46]. Besides, the *ERECTA* family is one of the master factors regulating epidermal stomatal formation. *Arabidopsis erecta* mutants showed a phenotype of increased stomatal density and higher water consumption [47]. However, whether MMCs can develop into guard cells or not depends on the signaling peptide *ERECTA* captured. Three peptides from the Epidermal Patterning Factor-Like (EPFL) family, *EPF1*, *EPF2*, and *EPF9* (*STOMAGEN*) as ligands, are involved in cell fate determination in the *ERECTA*-dependent pathway. *EPF1* and *EPF2* are secreted by stomatal lineage cells. After being captured by the LRR domain of *ERECTA*, the intracellular kinase catalytic domain of the receptor-ligand complexes activates the *MPK* cascade downstream by phosphorylation, which leads to the phosphorylation of *SPCH* and perhaps *MUTE* to regulate their transcription and activity. Consequently, this switches off the process of the stomatal lineage [20,48,49,50]. *EPF9*, oppositely, is produced by internal mesophyll cells and agonistically binds to *ERECTA* to block its kinase activity and triggers the formation of stomata [50,51,52,53,54]. This competition maintains a balance between CO_2_ assimilation and water consumption, which is essential for plants to survive in a fickle natural environment.

Forests help to maintain biodiversity, protect land and water resources, relieve climate change, and provide food and raw materials for human beings [55]. As one of the most valuable commercial tree species in many countries, the *Populus* species, which is widely planted in northern China, is the foremost fast-growing tree in the temperate region and of great value in afforestation, ecology, and landscape architecture. Along with its rapid growth rate, however, poplar plantation is quite water intensive, which makes it vulnerable to water deficiency and limits its cultivated area. As the world’s most populous country, China is confronting the shortage and maldistribution of freshwater, which makes it onerous to farm and afforest in almost half of the terrain of China, not to mention the growing populations, the development of industrialization and urbanization, and the deteriorating global climate, which is variable and unpredictable [56,57,58,59,60,61,62]. Under these conditions, it is urgent that trees’ water use is reduced whilst maintaining growth. In recent decades, a massive number of reports have proved that tree genetic engineering is an efficient way to improve the growth rate, wood quality, and stress tolerance in poplar [55,63,64,65]. Accompanied by conventional silviculture, it may be an efficient and prospective technic to meet the explosive demand for lumbers and release deforestation.

Previously, we cloned the homologous gene of *Arabidopsis ERECTA* from a high water use efficiency (WUE) poplar genotype NE-19, *Populus nigra ×* (*P. deltoides × P. nigra*), and the *PdERECTA* overexpression *Arabidopsis* plants showed a phenotype of a larger leaf area, higher inflorescence, stronger photosynthesis capacity, increased biomass accumulation, reduced stomatal density, and improved water use efficiency [66]. However, the performance of *PdERECTA* in responding to drought stress remains largely unknown. Besides, in contrast to a large number of reports on herbaceous plants concerning the *ERECTA* gene, only a limited number of studies have investigated the functions of *ERECTA* on woody plants. For these reasons, *PdERECTA* was introduced into a triploid white poplar, *P. tomentosa* ‘YiXianCiZhu B385’ [67], under the drive of CaMV35S via *Agrobacterium tumefaciens*-mediated transformation in this study. The purpose was to examine the functions of *PdERECTA* in poplar and assess the feasibility of tree breeding by genetic modification using *PdERECTA*.

## 2. Results

### 2.1. Expression Pattern of PdERECTA and Subcellular Localization

In *Arabidopsis*, *ERECTA* was expressed in shoot apical and organ primordia and hypocotyl [37,68]. In poplar, *PdERECTA* was reported to have high expression in young top stems and young leaves but not in roots [66]. However, the precise tissue expression pattern of *PdERECTA* in poplar remains unknown. In our research, we first analyzed the *cis*-elements of the *PdERECTA* promoter using the PlantCARE database (http://bioinformatics.psb.ugent.be/webtools/plantcare/html/, last accessed on 30 June 2021). A series of *cis*-acting elements involved in plant development were identified, including CAT-box (related to the meristem expression), HD-zip 1 (related to palisade mesophyll cell differentiation), and TGA element (related to auxin response) (Supplementary Materials Figure S1). Besides, there were 18 light-responsive elements presented in the promoter region, indicating that *PdERECTA* played a special role in plant development and photomorphism (Supplementary Materials Figure S1). Besides, there were several stress-related elements, such as ARE (responding to anaerobic) and LTR (responding to low temperature). The MeJA response element CGTCA-motif and TGACG-motif, salicylic acid response element TCA-element, and abscisic acid response element ABRE were also labeled in the promoter region.

To further analyze the expression pattern of *PdERECTA* in different tissues, the transgenic poplar seedlings containing pPdER::GUS were used for histochemical staining. Noticeably, *PdERECTA* is mainly expressed in buds, young organs, and leaf veins, and is highly accumulated in the petiole (Supplementary Materials Figure S2c). Besides, the activity was also detected in the roots, though not all of them (Supplementary Materials Figure S2b).

**Figure 1:**
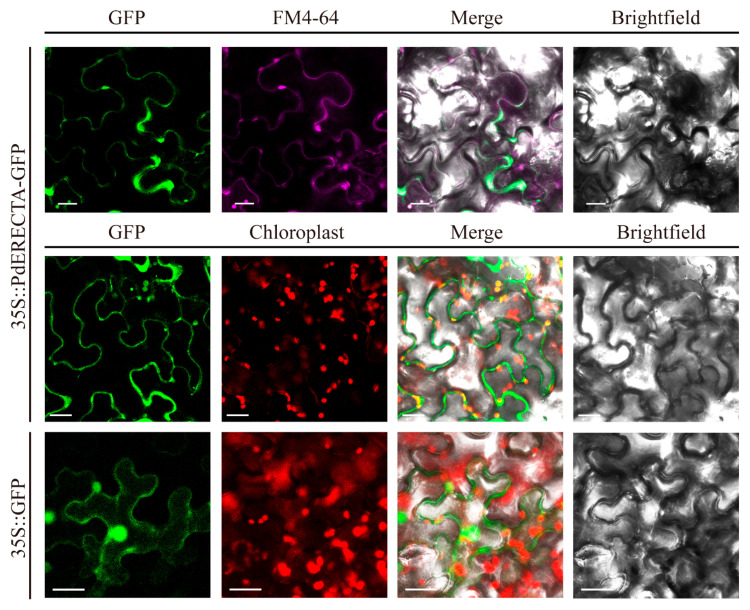
Subcellular localization of *PdERECTA*-GFP in transiently expressed tobacco leaves. FM4-64 was used to mark the plasma membrane. GFP without fusion with *PdERECTA* was also transiently expressed in tobacco under the drive of CaMV35S and used as a control. Bar = 20 µm.

The result of the subcellular localization assay showed that the GFP signal of *PdERECTA-*GFP fusion protein and FM4-64 was simultaneously detected at the periphery of epidermis cells (Figure 1), disclosing that *PdERECTA* localized to the plasma membrane.

### 2.2. PdERECTA-Modified Stomatal Density and Size in Poplar

To evaluate the functions of *PdERECTA* in poplar, we generated transgenic poplar plants overexpressing *PdERECTA*. Fourteen individuals (Ln1–Ln14) were confirmed by genome PCR analysis using *PdERECTA* and CaMV35S promoter-specific primers. The result of PCR showed expected bands (around 740 bp) in all these OxPdER lines and the positive control but not in the negative control and VT (Supplementary Materials Figure S3b). The qRT-PCR results further confirmed that *PdERECTA* was overexpressed successfully in all the OxPdER lines except VT. The relative expression levels of *PdERECTA* in these overexpression lines were around 2.5–20-fold of the VT line. Among all the OxPdER lines, Ln5 and Ln12 displayed the highest expression levels, with 21- and 17-fold that of the VT line, separately (Supplementary Materials Figure S3c). Ln5, Ln9, Ln10, and Ln12 were further propagated for physiological experiments.

**Figure 2:**
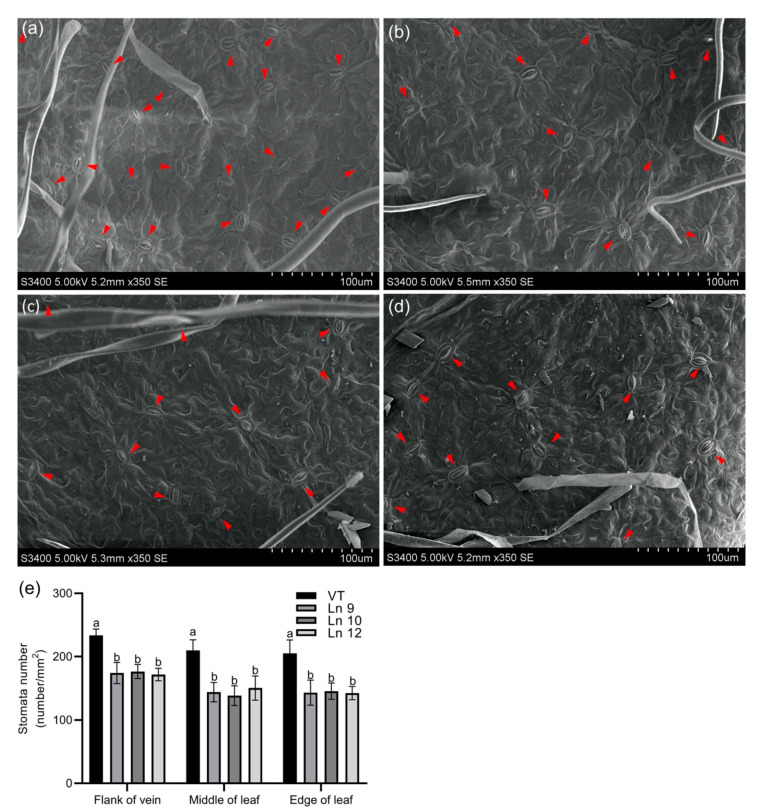
Leaf stomatal density in poplar. Scanning electron micrograph of the leaf of the VT control (**a**), Ln 9 (**b**), Ln 10 (**c**), and Ln 12 (**d**). The flank of the vein, middle of the leaf, and edge of the leaf were scanned and counted separately. Bar = 100 µm. (**e**) The stomatal density of leaf in VT and OxPdER. Data are mean value ± SE (*n* = 15). Duncan’s multiple range test (DMRT) was carried out to determine the significance among different lines. Means followed by different letters indicate significant differences at the *p* < 0.05 level.

**Figure 3:**
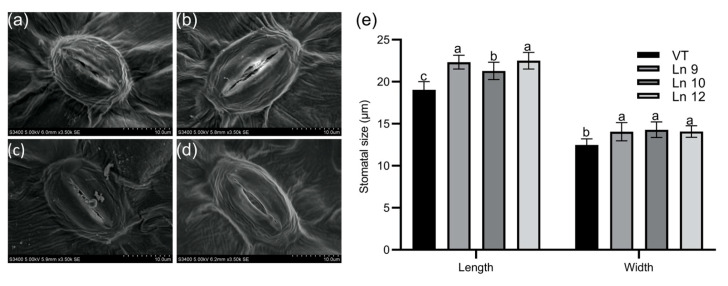
Stomatal size of the leaf in poplar. Scanning electron micrograph of the leaf of the VT control (**a**), OxPdER Ln 9 (**b**), Ln 10 (**c**), and Ln 12 (**d**). Bar = 10 µm. (**e**) The stomatal size of VT and OxPdER lines. Data are mean value ± SE (*n* = 15). DMRT was carried out to determine the significance among different lines. Means followed by different letters indicate significant differences at the *p* < 0.05 level.

Overexpressing *PdERECTA* in poplar dramatically reduced the stomatal density on the abaxial leaf surface. As shown in Figure 2, the stomatal number per unit area in the leaves of the OxPdER lines was 24–34% lower than that of VT. Meanwhile, the stomatal size was alternated in OxPdER lines (Figure 3). The stomata of OxPdER lines were enlarged compared with the VT line, being 11–18% longer than that of the VT line, and 12–14% wider than that of the VT line (Figure 3e).

### 2.3. Overexpressing PdERECTA in Poplar Improved Instantaneous Water Use Efficiency

**Figure 4:**
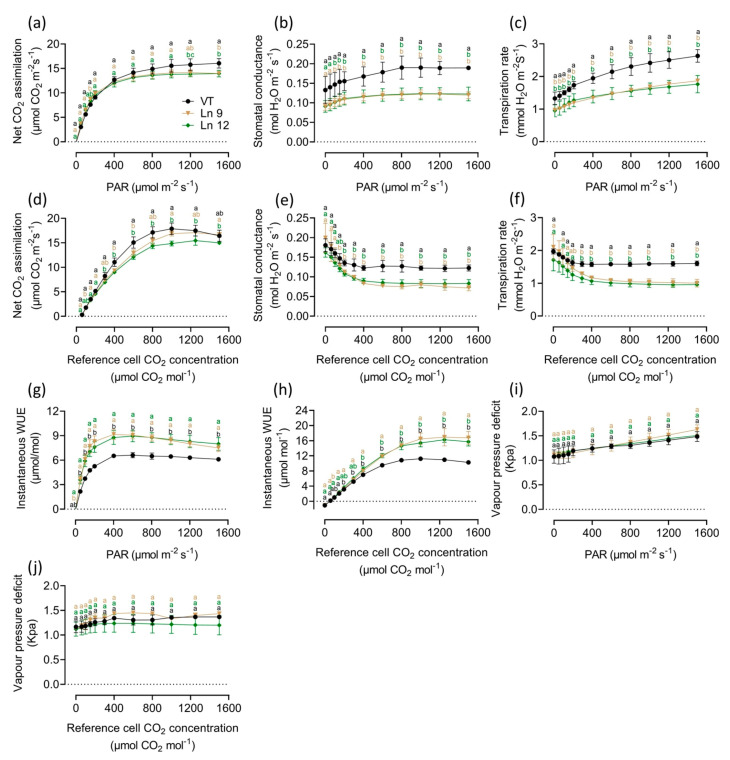
Light response and CO_2_ response curves of VT and OxPdER Ln 9 and Ln 12. These assays were taken in the same greenhouse in controlled environmental conditions. (**a**) Net CO_2_ assimilation-light curve. (**b**) Stomatal conductance-light curve. (**c**) Transpiration-light curve. (**d**) Net CO_2_ assimilation-CO_2_ curve. (**e**) Stomatal conductance-CO_2_ curve. (**f**) Transpiration-CO_2_ curve. (**g**) Instantaneous WUE-light curve. (**h**) Instantaneous WUE-CO_2_ curve. (**i**) VPD-light curve. (**j**) VPD-CO_2_ curve. Data are mean value ± SE (*n* = 3). DMRT was carried out to determine the significance among different lines. Means followed by different letters indicate significant differences at the *p* < 0.05 level.

Modification of the stomatal density and size directly impacts the gas exchange in leaves. As expected, the results of the instantaneous gas exchange assay showed significant differences between the OxPdER lines and the VT line. The photosynthesis–light and photosynthesis–CO_2_ curves indicated that the photosynthesis rate of the OxPdER plants was a little lower than that of the VT plants, which was about 10–14% under excess light and 3–19% under different CO_2_ concentrations (Figure 4a,d). However, the stomatal conductance of OxPdER plants was much lower than that of the VT line under both conditions (Figure 4b,e). Coming along with the reduction of stomatal conductance, the transpiration rate of OxPdER plants was much lower than that of VT, up to 32% and 35% when exposed to saturating light and CO_2_, separately (Figure 4c,f). Synthetically, the OxPdER plants had a higher WUE value compared with VT plants, which was around 27–38% higher when supplied with over 400 µmol m^−2^ s^−1^ PAR levels and around 14–63% higher when supplied with CO_2_ concentrations of over 400 µmol/mol (Figure 4g,h). Besides, the VPD value showed no significant difference under both conditions (Figure 4i,j), indicating that the reduction of transpiration was slightly affected by VPD.

### 2.4. PdERECTA Conferred Enhanced Short-Term Drought Tolerance to Poplar

**Figure 5:**
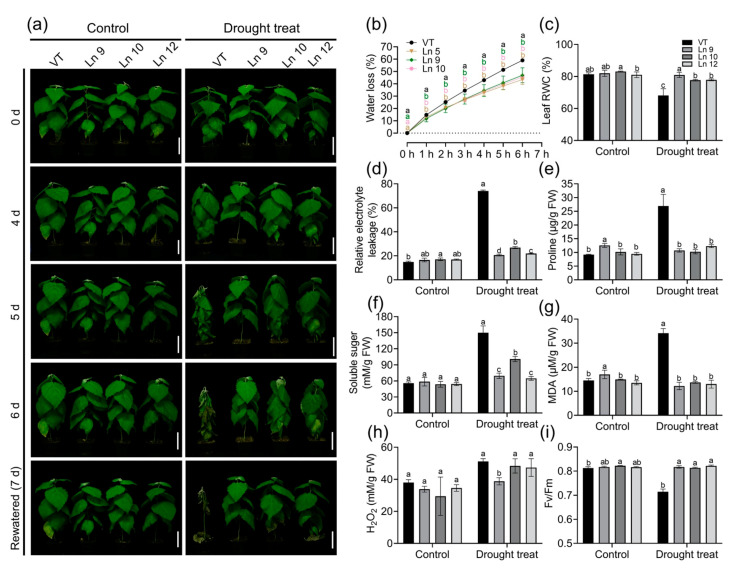
Overexpression *PdERECTA* in poplar exhibited enhanced drought tolerance in short-term drought stress. (**a**) Morphological differences in short-term drought stress. Bar = 10 cm. (**b**) Water loss of leaves. (**c**) Leaf RWC. (**d**) Relative electrolyte leakage. (**e**) Measurement of proline content. (**f**) Measurement of soluble sugar content. (**g**) Measurement of MDA content. (**h**) Measurement of H_2_O_2_ content. (**i**) The maximal quantum yield of PS II (Fv/Fm). Data are mean value ± SE (*n* = 3). DMRT was carried out to determine the significance among different lines. Means followed by different letters indicate significant differences at the *p* < 0.05 level.

As *PdERECTA* can reduce plant transpiration, we wondered whether it could improve tolerance of water deficiency in transgenic plants. The detached leaves of OxPdER plants showed a slower water loss rate than those of VT under natural dehydration (Figure 5b). To further observe the different abilities of the VT and OxPdER lines to endure drought stress, two-month-old plants were treated by withholding irrigation. On the fourth day, the leaves of the VT line began wilting, while the leaves of the OxPdER lines remained turgid (Figure 5a). On the sixth day, the plantlets of the VT line withered and the OxPdER lines began wilting. After being re-watered, the OxPdER lines returned to normal immediately while the VT line started leaf abscission. During this experiment, the plantlets of the control group grew normally, and no significance was observed between the VT line and OxPdER lines (Figure 5a).

Plants’ physiological changes directly reflects the influence of drought stress. The results showed that the RWC of OxPdER lines was significantly higher than that of VT after 5 days of treatment. The RWC of the VT line significantly dropped, from 81% to 68%, while the overexpression lines remained normal (about 77–80%) (Figure 5c). The REL of all the lines of the control groups was not significant, while the REL of the VT line increased significantly after drought treatment (from 15% to 74%), which was much higher than that of the OxPdER lines (around 20–27%) (Figure 5d). Proline and soluble sugar are important components for osmoregulation in plants. Under normal conditions, the proline content of the VT and overexpression lines was not significantly different, while after drought treatment, the proline content of VT increased significantly, from 9 to 26 μg/g, which was over two times higher than that of the OxPdER lines (10–12 μg/g) (Figure 5e). Similar to proline, the soluble sugar content rose in all lines after drought treatment, but it was higher in VT than that in the OxPdER lines (Figure 5f). Besides, the MDA content, an index of cytomembrane oxidative damage, was much higher in the VT line than that of the OxPdER lines after drought treatment, indicating serious cytomembrane damage in the VT line (Figure 5g). The H_2_O_2_ content was a little bit lower in the OxPdER lines in the control and treatment groups (Figure 5h). All these indices showed that VT suffered more severe drought stress compared with the overexpression lines.

**Figure 6:**
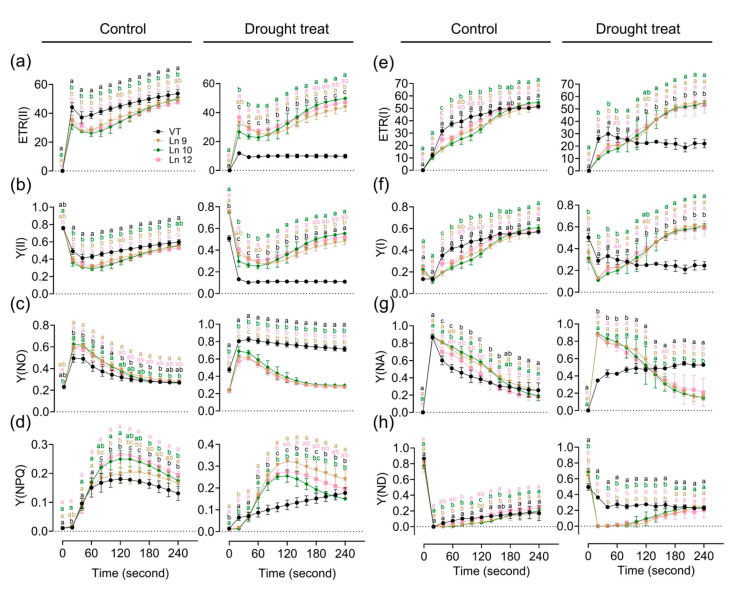
Impact of drought stress on photosynthetic activity and electron transport in poplar. Time-related photosynthetic parameters relative ETR(II) (**a**), Y(II) (**b**), Y(NO) (**c**), and Y(NPQ) (**d**) of the PSII system, and relative ETR(I) (**e**), Y(I) (**f**), Y(NA) (**g**), and Y(ND) (**h**) of the PSI system were analyzed. Data are mean ± SE (*n* = 3). DMRT was carried out to determine the significance among different lines. Means followed by different letters indicate significant differences at the *p* < 0.05 level.

Water deficiency can impact the photosynthesis system. The maximum photochemical efficiency of the PS II (Fv/Fm) in OxPdER lines was significantly higher than that of the VT line after drought stress (Figure 5i). Y(II) (quantum yields of PS II) and ETR(II) (apparent electron transport rates of PS II) were higher in the VT line than in the OxPdER lines at the beginning of the exposure to actinic light under normal conditions, but the gap slowly shrank as time went on (Figure 6a,b). Under water-deficient conditions, however, Y(II) and ETR(II) dropped dramatically in the VT line, while that of the OxPdER lines was slightly impacted by drought stress (Figure 6a,b). The Y(NPQ) (quantum yield of regulated energy dissipation in PS II) and Y(NO) (quantum yield of non-regulated energy dissipation in PS II) represent the regulated and nonregulated energy dissipation at PS II centers. Y(NPQ) and Y(NO) were higher in the OxPdER lines at the beginning of the measurement under normal conditions, after drought stress; however, Y(NO) was much higher in the VT line than in the OxPdER lines, and Y(NPQ) of the VT line was lower than that of the OxPdER lines, meaning that the VT line was suffering severe damage from excess light (Figure 6c,d). Electron transport through PS I is an important index to detect where the interruption of electron flow occurs. We further analyzed the PS I parameters obtained from the P700 signals. Y(I) (quantum yields of PS I) of the VT line was higher under normal conditions at the beginning of light induction, but there was no significant difference at the end of this assay (Figure 6f). After drought treatment, Y(I) of VT remained at a low level (Figure 6f). As a contrast, the OxPdER lines were only slightly impacted by drought stress (Figure 6f). Y(ND) is the nonphotochemical PS I quantum yield of donor-side limited heat dissipation, representing the limitation of electron flow from PSII toward PS I, and Y(NA) is the nonphotochemical PS I quantum yield of acceptor-side limited heat dissipation, representing the fraction of overall P700 that cannot be oxidized by a saturation pulse in a given state due to a lack of oxidized PSI acceptors [69]. Under drought stress, the Y(ND) of the VT line was higher at the beginning than that of the OxPdER lines and remained at a high level (Figure 6h). Similar to Y(ND), Y(NA) of the VT lines remained at a relatively high level during the assay, while it reduced gradually with the assay going on in the OxPdER lines (Figure 6g). These results indicated that the photosynthesis process of the VT line was interrupted after drought stress.

### 2.5. PdERECTA Conferred Enhanced Drought Tolerance to Poplar under Long-Term Water Deficit

**Figure 7:**
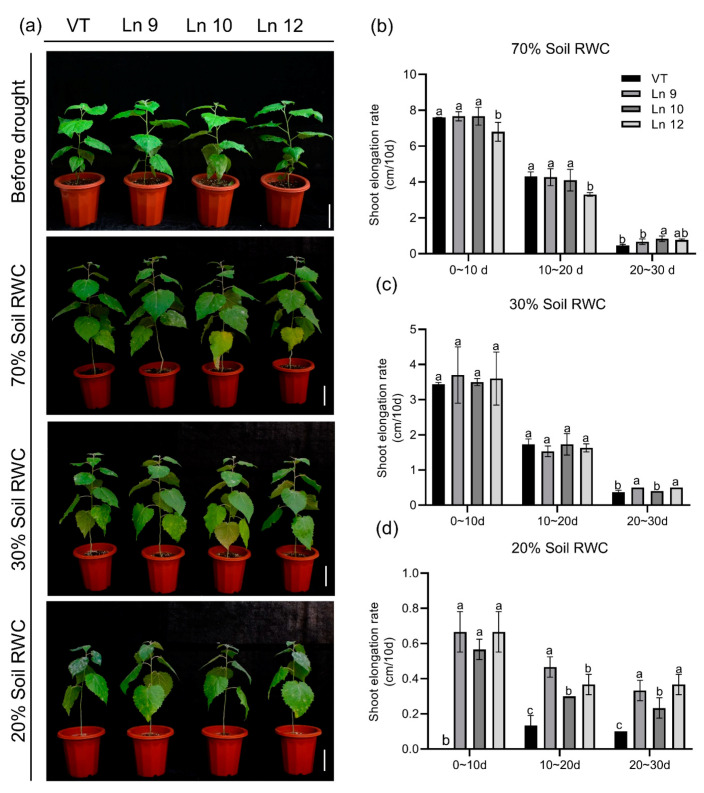
Overexpressing *PdERECTA* in poplar exhibited better endurance to long-term water deficit than control. (**a**) Morphological differences in long-term drought experiments. Bar = 10 cm. (**b**–**d**) Growth rate under normal conditions (**b**), middle water deficit (**c**), and severe water deficit (**d**). Data are mean value ± SE (*n* = 3). DMRT was carried out to determine the significance among different lines under different treatments. Means followed by different letters indicate significant differences at the *p* < 0.05 level.

**Figure 8:**
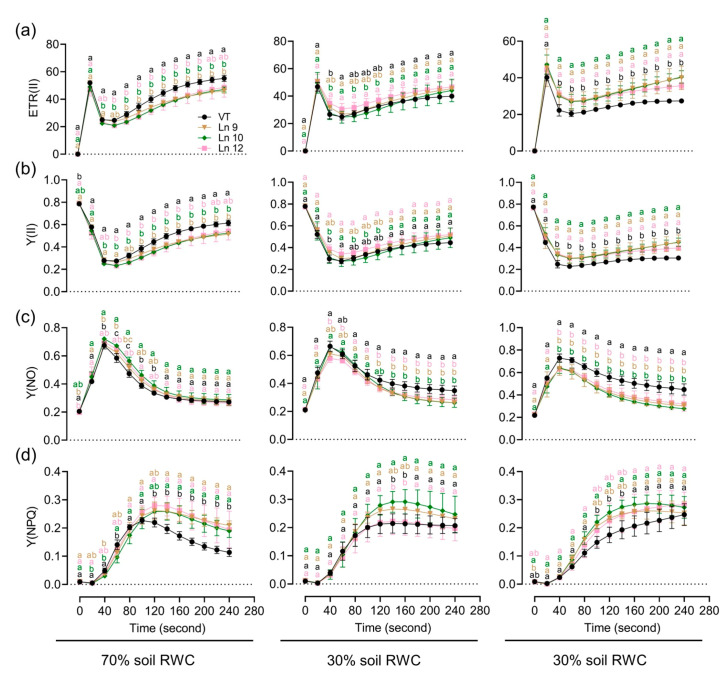
Impact of a long-term water deficit on the photosynthetic activity and electron transport of PSII in poplar. Time-related photosynthetic parameters relative to ETR(II) (**a**), Y(II) (**b**), Y(NO) (**c**), and Y(NPQ) (**d**) of the VT and OxPdER under normal, middle, and severe drought stress are shown. Data are mean value ± SE (*n* = 3). DMRT was carried out to determine the significance among different lines under different treatments. Means followed by different letters indicate significant differences at the *p* < 0.05 level.

To test if the OxPdER transgenic poplar plants showed altered long-term drought tolerance, we further explored their performances under a long-term water deficit environment. Similar plants of the VT line and OxPdER lines were exposed to 40-day drought treatment. However, the VT line showed a weaker performance, especially at 20% soil RWC (Figure 7a). There was no significant difference in the growth rate under normal and middle water deficit conditions among all these lines; however, the VT line could barely grow under severe drought stress conditions, while the OxPdER lines showed higher growth rates than the VT line (Figure 7b–d). The results of the chlorophyll fluorescence showed that the OxPdER lines had higher electron transport rates [ETR(II)] and yields of PS II [(Y(II)] under drought stress, indicating that the OxPdER lines had higher photosynthetic ability (Figure 8a,b). The Y(NO) of the VT line was higher than that of the OxPdER lines under drought stress conditions, and Y(NPQ) was lower (Figure 8c,d), meaning that the VT line was more sensitive to excess light. These results showed that the OxPdER lines had enhanced photosynthesis ability under water deficit conditions.

**Figure 9:**
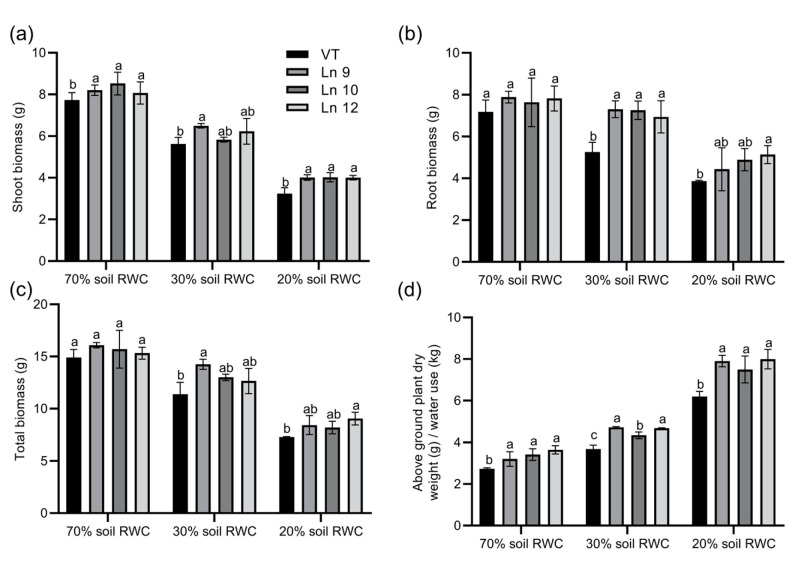
Biomass accumulation of VT and OxPdER lines under a long-term drought deficit. (**a**) Biomass of shoot. (**b**) Biomass of root. (**c**) Total biomass (**d**) Integral WUE during the treatment. Data are mean value ± SE (*n* = 3). DMRT was carried out to determine the significance among different lines under different treatments. Means followed by different letters indicate significant differences at the *p* < 0.05 level.

Next, we monitored the differences in total biomass accumulation and WUE among OxPdER and VT lines. Data showed that the shoot biomass accumulation and root biomass accumulation were higher in OxPdER lines under severe drought stress, which was up to 39% and 25%, separately (Figure 9a,b). In total, the biomass of the OxPdER lines was higher than that of the VT line when subjected to long-term drought stress (Figure 9c). Synthetically, the OxPdER lines exhibited a higher long-term WUE than VT at all water levels, which were around 17–33% higher at 70% soil RWC, 18–28% at 30%, and 20–28% at 20% (Figure 9d). All these results confirmed that overexpressing *PdERECTA* in poplar improved plant WUE and assisted plants to endure drought stress, especially severe drought stress.

## 3. Discussion

RLKs are proteins that act as upstream signaling components controlling massive essential processes [70]. It has been known that the protein encoded by the *Arabidopsis* gene *ERECTA* is one of the LRR-RLK proteins that regulates multiple signaling pathways [71]. Despite this, the in vivo role of the *AtERECTA* orthologs in woody plants is barely known. We previously analyzed a homolog gene of *Arabidopsis ERECTA* gene, *PdERECTA*, from *Populus.* The bioinformatics analysis of the amino acid sequence encoded by *PdERECTA* showed that *PdERECTA* consists of an LRR extracellular domain, a transmembrane region, and a cytoplasmic serine/threonine (Ser/Thr) protein kinase domain, a typical structure of LRR-RLKs, and overexpressing *PdERECTA* can finely complement the *ERECTA* gene nutant phenotype in *Arabidopsis* [66]. Due to the high conservation of *ERECTA* genes across species [72], we hypothesized that *PdERECTA* might regulate drought tolerance in poplar. In this study, we provided some evidence of its capacity in regulating stomatal abundance in leaves and drought resistance.

It is well known that *ERECTA* signaling is associated with vegetative growth and development and inflorescence [38,39,43,73], plant immunity [74,75,76], and other stimulation of abiotic stress [77,78,79]. In this research, the element analysis of the promoter region of *PdERECTA* showed that the *PdERECTA* promoter contains several stress-responsive elements. The result of the GUS staining in poplar showed that the *PdERECTA* was mainly expressed in stems and buds, which is consistent with a previous report [37], and the high expression level in the petiole reflects the important function in shade avoidance in responding to light fluctuation [68,77,80]. The *ERECTA* family is predicted to localize to the plasma membrane in *Arabidopsis* [36]. Similar to *Arabidopsis*, *PdERECTA* precisely localizes to the cell plasma membrane, where it transduces signals from the environment [35].

Stomatal density in leaves is determined by the regulation of guard cell differentiation, the asymmetric division of guard cell meristemoids, or epidermal cell expansion [81,82]. Stomatal density is affected by the number of stomata and the size of epidermis cells [83]. Overexpressing *PdERECTA* in poplar can alter established stomatal development and patterning, manifested as reduced stomatal density and enlarged pavement cells and guard cells, resembling the phenotypes observed before [18,84]. These phenomena might be proof of the thesis that stomatal density is negatively correlated with stomatal size [25,85], which is proven to be a mutual complement mechanism to maximize the assimilation of CO_2_. New evidence has given a hint to explain these phenomena. As reported, *AtEDT1/HDG11* can regulate the expression levels of *ERECTA* and thus modulate the cell cycle through interaction with *E2Fa*, which can, at last, contribute to the increased cell size and decreased stomatal density [86]. As *ERECTA* takes part in the synthesis and transportation of auxin to control cell elongation [87] or repressing the sensitivity to cytokinin [88], and loss of function of *ERECTA* leads to a low cell expansion rate in all zones of the leaf and all successive leaves of a plant [89], the reduced stomatal density and enlarged cell size may be a combination of enhancing cell expansion, restricting cell division, and preventing the transformation from MMC to GCs.

Reduced stomatal density affects water and CO_2_ exchange [25]. Moreover, one study reported that stomatal size has a key role in water movement from soil to leaves and is negatively correlated with drought stress tolerance [90]. Thus, how to balance CO_2_ assimilation and water evaporation is an intricate task to improve WUE by manipulating stomatal density. The influences of reduced stomatal density on gas exchange and photosynthesis were reflected in our study. This reduction led to a dramatic decrease of stomatal conductance in OxPdER, which resulted in a distinct restriction of water evaporation. As a consequence, the instantaneous WUE was much higher in *PdERECTA* overexpression lines. The same phenomena were observed in *Arabidopsis* [17,91], *Populus* [92], and rice [19]. This disproportion, mainly due to the diffusion rate of water vapor, is greater, about 1.6 times that of CO_2_ [25]. 

Water deficit affects many metabolic processes, including photosynthesis, and damages basic organization structure and photosynthetic apparatus, which inhibits the assimilation of carbon, leading to a decreased yield [93,94]. Manipulation of stomatal density has been proven to be a potential tool to improve drought stress tolerance with little effect on nutrient uptake [95]. In our study, the leaf dehydration shock test demonstrated that the reduction of the stomatal abundance in the leaves of the OxPdER plants can efficiently slow down dehydration. Under sudden drought stress, the OxPdER plants exhibited a stronger water retention capacity. These phenomena showed that poplars with reduced stomatal density might have some advantages growing in a water-deficient area. This has been proven to be a powerful strategy to avoid dehydration when confronted with drought stress. As positive regulators of *ERECTA*, the *AtEDT1/HDG11* overexpressing plants showed reduced stomatal density and enhanced drought and osmotic stress tolerance [96]. A similar phenotype was found while overexpressing *AtEDT1/HDG11* in rice [97]. Overexpressing a ligand of *ERECTA*, *PdEPF1*, in poplar showed substantially reduced stomatal abundance and enhanced endurance to a short-term and long-term water deficit [18]. One report further showed that perpetually overexpressing *OsEPF1* in rice only consumed 60% of the normal amount of water wild-type plants consumed between weeks 4 and 5 post germination [19]. Consistent with the *ERECTA-*dependent pathway, overexpression of a *SchSDD1*-like gene from wild tomato slowed down the water loss rate of plants [15]. The *AtTGL1* loss-of-function mutations showed reduced stomatal density, lower transpiration, and improved drought tolerance [98]. One miRNA from *P. ussuriensis*, *Pu-miR172d*, can cleave its target *PuGYL1*, upregulating the expression of *PuSDD1*, and this alteration resulted in reduced stomatal density and enhanced drought stress endurance in poplar [92]. Prolonged exposure to drought stress leads to the destruction of the photosynthetic apparatus and inhibition of photosynthesis in plants [99,100]. The results of the chlorophyll fluorescence showed that the photosynthesis system was broken down and excess light energy cannot be dissipated via nonphotochemical quenching in the VT line after short-term drought stress. This collapse of photosynthesis and the self-protecting mechanism means a loss of the ability to deliver and fix or dismiss the light energy leaf captured, and this excess energy becomes a menace to the cell structure [101]. A similar phenotype was observed when plants were subjected to a long-term water deficit. As a consequence, OxPdER lines had a higher growth rate and biomass accumulation, especially under severe drought conditions. Taking all these results together, we conclude that poplar plantlets with reduced stomatal abundance have better endurance during a water deficit.

Though a significant reduction of the transpiration rate and dramatically improved WUE was confirmed in OxPdER lines, the decline of CO_2_ assimilation could not be neglected. To ensure maximum leaf diffusive (stomatal) conductance of CO_2_ for photosynthesis to counter the low atmospheric CO_2_ concentration in the process of involution, the strategy plants have evolved is a higher density instead of a larger stomatal size [25,85]. Otherwise, increasing transpiration is beneficial to nutrient uptake when supplied with plentiful water [95]. There is concern about whether a reduced number of stomata affects plants’ photosynthesis ability. Although it is reported that decreased stomatal density has no effect on photosynthesis for the complementation of enlarged stomata [102], and an enlarged stomatal size was also observed in the *PdERECTA* overexpression line in our study, a reduced number of stomata does impact the diffusion of CO_2_ into the leaf when supplied with unsaturated CO_2_ concentrations. The same phenomenon was found in rice with reduced stomatal density [19]. We normally associate this with the impact of stomatal density reduction, but a slight difference in the assay of chlorophyll fluorescence between the VT line and OxPdER lines was detected in our study. Under normal growth conditions, the quantum yield and electron transport rates were all a little bit lower in the OxPdER lines than that in the VT line, at least at the beginning of the photosynthesis process. It seemed to be a little slower at responding to light after dark adaption in the OxPdER lines, indicating a little altered photosynthesis ability. This alteration might partly be due to the reduced CO_2_ assimilation, thus resulting in a lack of adequate zymolyte to fix light energy, but further elucidation of the precise mechanisms underlying *ERECTA* affecting the photosynthesis system should be a focus of future work.

Pathogens cause numerous diseases, especially for plants, most of which are growing in soil. Plant diseases cause numerous economic losses each year around the world. Many genes take part in plant immunity, including LRR-RLKs [35]. As reported, *ERECTA* is a vital gene involved in the host defense against pathogens, both bacterial and fungus. The *Arabidopsis* plants transformed with the *ERECTA* gene showed an increased tolerance to bacterial wilt [74]. The *Arabidopsis erecta* mutant alleles are more susceptible to *Plectosphaerella cucumerina* [103]. Further research demonstrated that *BAK1* can interact with *ERECTA* and *TMM*, as the multiproteic receptorsome formed by the three genes modulates *Arabidopsis* resistance to this pathogen [76]. One of the strategies might be regulating cell wall construction. The cell walls of the *erecta* mutants showed reduced neutral sugars and increased uronic acids [104]. The downstream gene of *ERECTA*, *YODA MAP3K kinase*, is involved in the *ERECTA*-depended signaling pathway responding to pathogen [105] and virus infection [106]. In our research, we noted that the VT line was more vulnerable to fungus infection, especially when subjected to a water deficit (Figure 7a). Apart from the roles *ERECTA* has in the plant immune response, this difference might partly be due to the VT line suffering from severe drought stress, which made it more sensitive to fungus in the worse physiological conditions, as there was little difference in the 70% soil RWC group. However, the mechanism of *ERECTA* in the plant immune response requires further examination.

*Populus* species are the most important fast-growing trees in the temperate region used for timber and sand fixation. As half of the terrain of China lacks water, it is an onerous, perpetual, but meaningful task to cultivate water-saving and stress-resisting tree species to improve land use efficiency. In this study, we demonstrated that *PdERECTA* can regulate stomatal development and patterning in poplar, as the stomatal number was decreased while the size of the stomata was enlarged in *PdERECTA* overexpression plants. These modifications led to declined stomatal conduction and transpiration, and dramatically improved WUE, without sacrificing the carbon uptake potential too much. *PdERECTA* overexpression plantlets showed an improved ability to endure a short-term and long-term water deficiency. These traits might enhance the survival rate of poplar plantlets in a difficult site where fresh water is lacking. According to these results, we conclude that *PdERECTA* is a promising candidate gene for poplar genetic modification to breed water-saving and drought-tolerating tree species.

## 4. Materials and Methods

### 4.1. Plant Materials and Growth Conditions

The stem cuttings of a one-year-old poplar genotype NE19 were collected in April and planted in soil in an outdoor nursery of Beijing Forestry University, Beijing, China (40°000′ N, 116°200′ E; 49 m above sea level). Three-month-old plantlets were used for RNA extraction.

Triploid white poplar ‘YiXianCiZhu B385’ was used for genetic transformation. Aseptic plants were cultured in a mericlone nursery, which supplied a 16/8 h (light/dark) photoperiod and 28 °C room temperature. Sterilized leaves and stems were laid on solid Differentiation Medium [Murashige and Skoog (MS) medium, 0.1 mg/L a-naphthalene acetic acid (NAA), 0.02 mg/L thidiazuron (TDZ), 3% (*w*/*v*) sucrose, and 0.6% (*w*/*v*) agar] for adventitious shoot generating. The medium was refreshed every two weeks until the adventitious shoots were around 1 cm. Regenerated shoots were then cut and transferred to a Root Inducing Medium (RIM) [half strength MS medium, 0.05 mg/L NAA, 3% (*w*/*v*) sucrose, and 0.6% (*w*/*v*) agar]. All the mediums were adjusted to a pH value of 5.8 with 1 M NaOH. Rooting plantlets were cultured and propagated in RIM.

Two-month-old tissue-cultured plantlets were gently moved out and washed to remove solid medium and then planted into potting soil and covered with transparent plastic films and then moved into a plant growth chamber. The growth conditions were 25 °C, around 60% relative humidity, and a 14 h photoperiod. These plantlets were watered every four days. For further assay, the grown-up plants were then transferred into a plastic greenhouse and grown under natural light.

### 4.2. Reverse Transcription and qRT-PCR

Leaves samples were quickly frozen by liquid nitrogen after being excised from plantlets and ground in liquid nitrogen. Total RNA of each sample was extracted using RN33-PLAN Tpure Plant Total RNA Extracting Kits (Aidlabs Bio Inc., Beijing, China) according to the manufacturer’s specification. The quantity and quality were determined as described before [107], and the first-strand cDNA was synthesized from 2 μg RNA using the Quant One Step RT-PCR kit (TianGen Bio Inc., Beijing, China). The reverse transcription products were then diluted into around 100 ng/μL and used for quantitative real-time PCR using SuperReal Pre Mix Kits provided by TianGen Bio Inc. (Beijing, China). Then, 18S ribosomal RNA was used as the endogenous reference gene. The relative expression level was calculated by the 2^−ΔΔCT^ method. The StepOne Plus Real-Time PCR System (Applied Biosystems, Inc., Carlsbad, CA, USA) was applied in our study.

### 4.3. Gene Cloning and Vector Construction

The cDNA sequence of *PdERECTA* (Accession number in GenBank: HM775855) was amplified by PCR from the total cDNA of poplar NE-19 and cloned into the pCAMBIA-1301 binary vector driven by the CaMV-35S promoter.

Genomic DNA was extracted from leaves by the CTAB method using DN14-CTAB Plant Genomic DNA fast extracting kits (Aidlabs Bio Inc., Beijing, China) following the manufacturer’s procedure. The promoter region around 3.0 kb upstream of the *PdERECTA* translation initiation site was cloned from the genome DNA of NE19 and constructed into the pCAMBIA1391 vector to drive the GUS (β-glucuronidase) reporting gene (marked as pPdER::GUS). All these reconstructed plasmids were then introduced into the *A. tumefaciens* strain EHA105.

### 4.4. Subcellular Localization

The ORF of *PdERECTA* was fused with GFP (green fluorescent protein) and ligated into the pCAMBIA1301 vector driven by the CaMV-35S promoter and then introduced into the *A. tumefaciens* strain GV3101. GFP that was not fused with *PdERECTA* was also cloned into the pCAMBIA1301 vector under the drive of CaMV-35S and introduced into *A. tumefacien*. *A. tumefaciens* containing these reconstructed vectors were infiltrated into the leaves of tobacco (*Nicotiana benthamiana*). The infiltrated tobacco plants were then incubated in the dark for 48 h. The infected leaves were then immersed in the solution containing FM4-64 dye [*N*-(3-Triethylammoniumpropyl)-4-(6-(4-(Diethylamino) Phenyl) Hexatrienyl) Pyridinium Dibromide, a plasma membrane-specific dye] and observed using a laser confocal fluorescence microscopy (ZEISS LSM780; Zeiss, Oberkochen, Germany). A 488 nm argon laser was used to excite GFP and FM4-64. Emissions were collected over a wavelength range of 492 to 545 nm for GFP, and 620 to 700 nm for FM4-64, separately.

### 4.5. Generation of Transgenic Populus plants

One- to two-month-old plantlets growing in culture bottles were used for genetic transformation. The procedure was described before with a few modifications [18,108,109,110]. The impaired leaves were dipped into the infection medium, which contained the *A. tumefaciens* incubated in YEB medium overnight till the OD_600_ = 0.4–0.6, for around 8–10 min with gentle shaking. The infected tissues were then transferred into differentiation medium and incubated in the dark for two days. After, transfected leaves were washed 3–4 times with sterile water supplemented with 500 mg/L cefotaxime and transferred to the selective medium (differentiation medium plus 300 mg/L cefotaxime and 5 mg/L hygromycin). Resistant vegetative propagules on the selective medium were cut and transferred into RIM plus 300 mg/L cefotaxime and 5 mg/L hygromycin for further screening. Empty vector without the *PdERECTA* coding sequence was also induced into *A. tumefaciens* and went through all these processes. The grown-up plantlets were propagated and then transplanted into pots.

### 4.6. Histochemical Staining

GUS activity detection was performed by histochemical staining. Fresh poplar leaves or plantlets were submerged in GUS reaction buffer then incubated at 37 °C for 12 h. Stained samples were discolored using 75% alcohol 3–4 times and then photographed.

### 4.7. Molecular Verification

For DNA analysis, the purified genome DNA extracted using the CTAB method from both the vector control line (VT) and *PdERECTA* overexpression lines (OxPdER) used for PCR using the vector-specific primers.

For RNA analysis, the leaves of VT and OxPdER lines were collected and frozen in liquid nitrogen and used for RNA extraction. The relative expression level of *PdERECTA* in both the VT and overexpression lines was detected by qRT-PCR.

### 4.8. Stomatal Density and Size Determination

The leaf samples of the flank of leaf vein, middle of the leaf, and the edge of the leaf were collected separately from fully expanded leaves using a punch (6 mm in diameter), and over 10 leaves from 5 plantlets each line were used in this assay. To measure the stomatal size, the leaf samples were immersed in 10 μM/L ABA solution and kept in the dark for two hours before fixation. All these samples were fixed, dehydrated, and examined as described before [18]. The abaxial leaf surface of each sample was observed using a scanning electron microscope (Hitachi S-3400 N, Chiyoda-ku, Tokyo, Japan). The magnifications used for stomatal density analysis and stomatal size analysis were 350 (with a visual field around 0.363 mm × 0.231 mm) and 3500 (with a visual field around 36 µm × 25 µm), separately. Three fields of view were taken in each sample, and over 6 samples were observed in each group. The number of stomata in each view was counted separately and the stomatal density was calculated as (number of stomata)/(0.363 mm × 0.231 mm). The size of the stomata was measured using Image-Pro Plus.

### 4.9. Leaf Instantaneous Gas Exchange Analysis

Two-month-old plantlets were used for the leaf instantaneous gas exchange analysis under the normal conditions in the plant growth chamber. Three lines, including VT and two OxPdER lines, were measured and each line contained five individuals. A Li-Cor portable photosynthesis analysis system (Li-COR 6400; Lincoln, NE, USA) was used to detect the net CO_2_ assimilating rate, transpiration, and stomatal conductance of the mature leaves of VT and OxPdER plants. The light and CO_2_ curves of the fully expanded leaves (the sixth to eighth leaf) were obtained using the internal programs in the Li-COR 6400 portable photosynthesis analysis system. Light curves were measured at photosynthetically active radiation (PAR) levels of 1500, 1200, 1000, 800, 600, 400, 200, 150, 100, 50, and 0 µmol m^−2^ s^−1^ with 500 µmol/mol external CO_2_. CO_2_ curves were measured at external CO_2_ concentration levels of 1800, 1500, 1250, 1000, 800, 600, 400, 300, 200, 150, 100, 50, and 0 µmol/mol with 800 µmol m^−2^ s^−1^ PAR. The experiment covered net CO_2_ assimilation, stomatal conductance, transpiration, and vapor pressure deficit (VPD). Instantaneous WUE was calculated as net CO_2_ assimilation/transpiration.

### 4.10. Short-Term Drought Treatment

Two-month-old plantlets growing in soil were used for the short-term drought stress assay in the plant growth chamber. All the plantlets of each line were divided into two groups, one for drought treatment and the other as the control. The soil was saturated with water and drained for 2 h before the treatment. All the plantlets of the drought treatment group stopped watering after that until day 7, while the control group was watered normally. On day 5, all the plantlets were used for physiological analysis. Phenotypes of the VT and OxPdER lines were photographed on the first day, fourth day, fifth day, and sixth day after stopping watering, and the seventh day after re-watering.

### 4.11. Long-Term Drought Experiment

Two-month-old plantlets grown in soil were used for the long-term water deficit assay. Fifty-eight plants of the VT and OxPdER lines were divided into three groups and the soil relative water content was kept at 70% (control), 30% (middle stress), and 20% (severe stress) separately. The soil RWC was calculated as (fresh weight − dry weight)/(saturated weight − dry weight) × 100%. Each line in each group contained five individuals, and each group contained three pots without plants to determine the soil evaporation. All the pots were weighed every day and supplemented lost water. Plant daily water consumption was calculated as supplemented water minus soil evaporation. This assay lasted for 40 days. The height of each plant was measured every five days. After 40 days, the chlorophyll fluorescence and plant biomass were determined.

### 4.12. Physiological Analysis

The fourth to sixth leaves were collected from the drought treatment group and control group separately for physiological analysis. To measure the leaf relative water content (RWC), the leaves were weighted immediately after being detached from plantlets, then stoved and weighted again.

The malondialdehyde (MDA) content was measured by thiobarbituric acid (TBA)-reactive substances. In total, 0.1 g of fresh leaves were ground with 2 mL of 10% trichloroacetic acid (TCA) then centrifuged at 4000 RPM for 10 min. Then, 0.5 mL of supernatant of each extracting solution were taken and mixed with 0.5 mL of 0.6% TBA and bathed in boiling water for 15 min. The reaction mixture was centrifuged after cooling down. The absorbance of the supernatant was measured at 532, 600, and 450 nm by a microplate reader (Tecan Infinite M1000 PRO, TECAN, Männedorf, Switzerland). The MDA content was calculated by the following equation: MDA (1 mol/g FW) = (6.45 × (A532 − A600) − 0.56 × A450) × Vr ÷ (Vs × FW) × Vt [Vt: Total volume of extract (mL); Vr: reaction volume (mL); Vs: Extract volume used for reaction (mL); FW: Sample fresh weight (g)]. All the experiments had three technical replicates.

To measure the leaf relative electrolyte leakage (REL), mixed leaf samples (0.1 g) of each line were washed gently with double distilled water three times, then immersed in 5 mL of double distilled water, and placed at room temperature for 3 h. The conductivity of the solution was detected using a DDS-307 Conductivity Meter (LEICI Company, Shanghai, China) and recorded as R1. All the samples were boiled for 15 min and then cooled down to room temperature. The conductivity of each sample was remeasured and recorded as R2. Blank double distilled water was used as a control to elucidate the innate conductivity and recorded as C1, C2, separately. The relative electrolyte leakage was calculated as REL = (R1 − C1)/(R2 − C2) × 100%.

The proline content, H_2_O_2_ content, and soluble sugar content were measured with the proline assay kit, hydrogen peroxide assay kit, and plant soluble sugar content test kit (Nanjing Jiancheng Bioengineering Institute, Nanjing, China) separately following the manufacturer’s instructions.

### 4.13. Chlorophyll Fluorescence and P700 Absorption Measurement

Photosynthetic activities were measured using a Dual-PAM-100 fluorometer (Walz, Effeltrich, Germany). All the plantlets were kept in the dark for 15 min before the assay. Chlorophyll fluorescence was measured and calculated as described before [111,112,113]. Actinic light (AL) was set to 214 mmol photons m^−2^ s^−1^ in this assay, and five individuals were measured every line in each group.

### 4.14. Statistical Analysis

The data are presented as the mean values ± SEs (standard errors). All the experimental data were analyzed with Statistical Product and Service Solutions 25.0 (SPSS, IBM, Armonk, NY, USA). One-way ANOVA was used to compare the statistical difference in the mean among the plant lines under different treatments based on Duncan’s Multiple Range Test (DMRT) at a significance level of *p* ≤ 0.05.

## Data Availability

All relevant data are within the paper and its Supplementary Materials.

## Supplementary Materials

The following are available online at https://www.mdpi.com/article/10.3390/ijms22147245/s1.
