# Supplementary figures and images for: The Receptor-Like Kinase ERECTA Confers Improved Water Use Efficiency and Drought Tolerance to Poplar via Modulating Stomatal Density
Source: Int J Mol Sci. 2021 Jul 6;22(14):7245. doi: 10.3390/ijms22147245 (PMC8303786; doi:10.3390/ijms22147245)

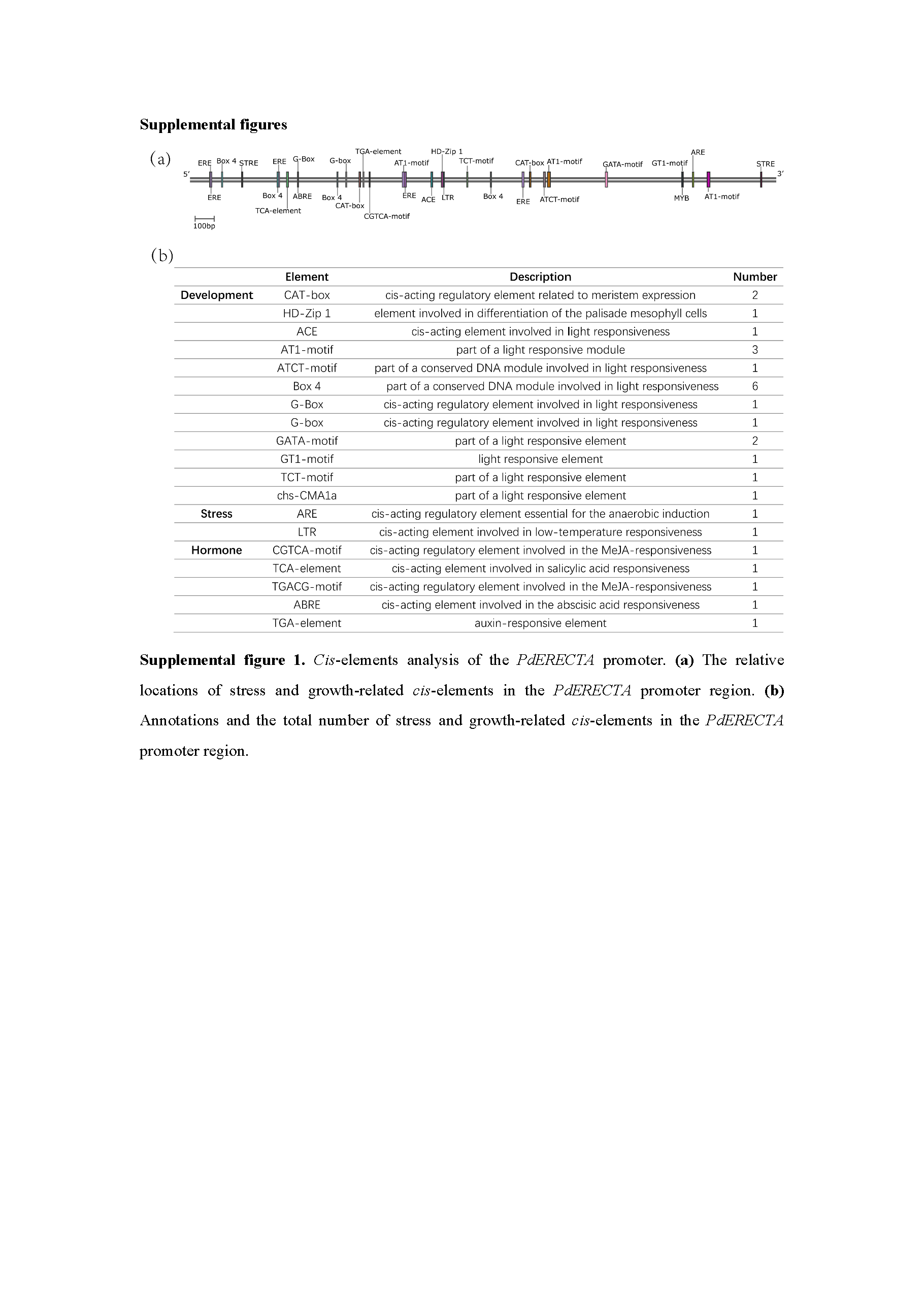

Supplement: Supplementary file 1 [file ijms-22-07245-s001.zip › Supplemental figures 1.tif]

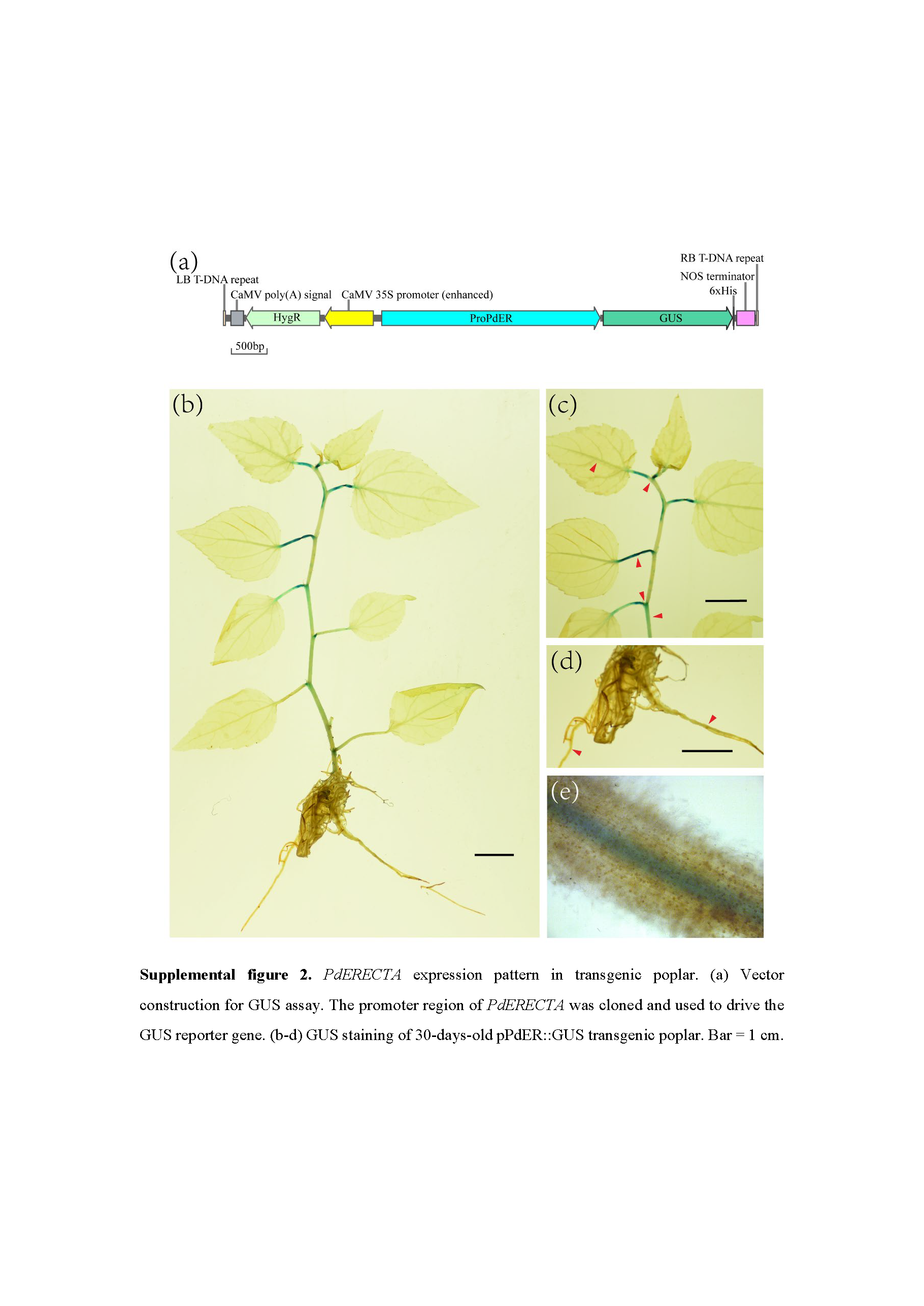

Supplement: Supplementary file 1 [file ijms-22-07245-s001.zip › Supplemental figures 2.tif]

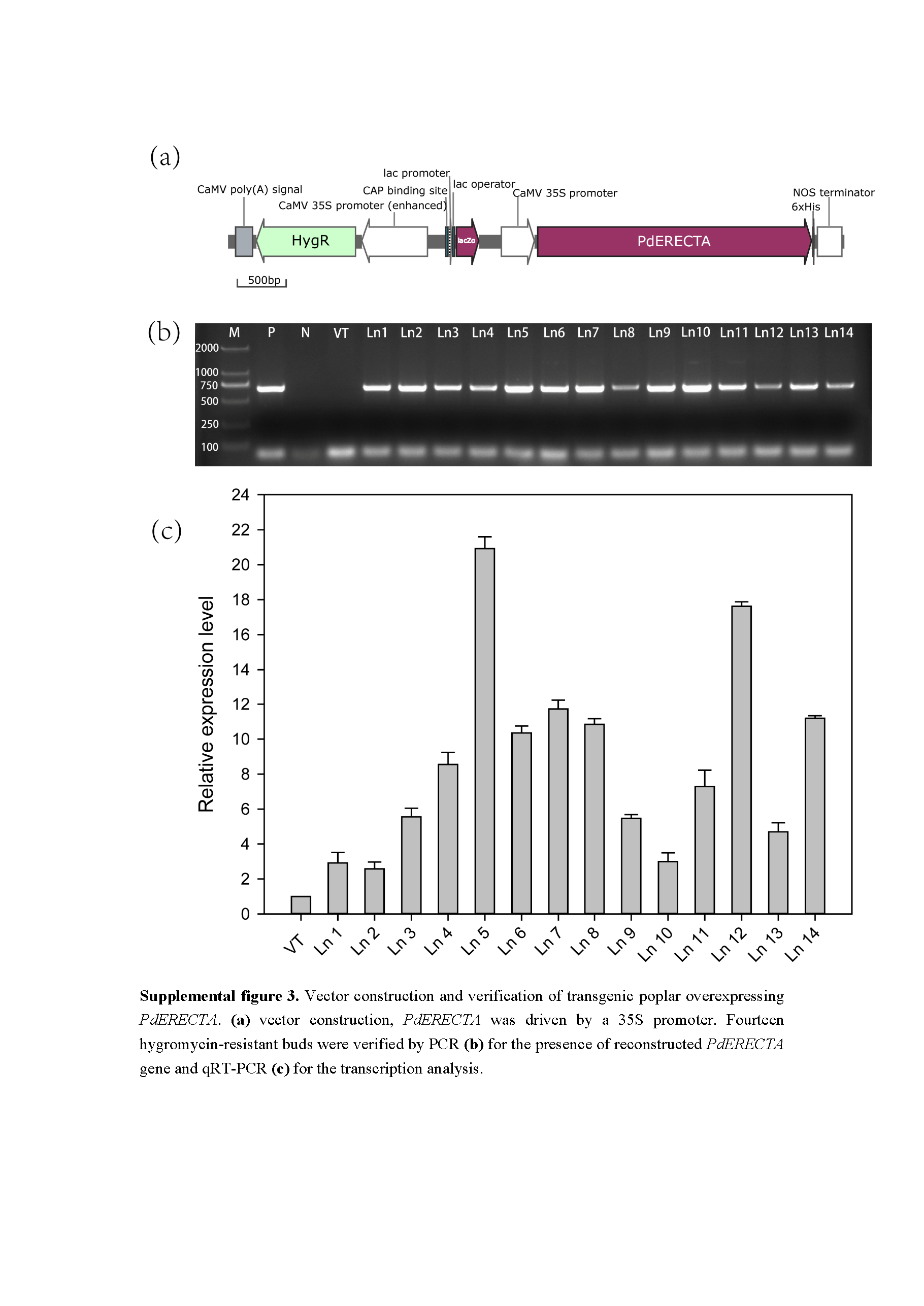

Supplement: Supplementary file 1 [file ijms-22-07245-s001.zip › Supplemental figures 3.tif]
